# 11492 Mapping CTSA hub activities across the EQ-DI framework to discover opportunities for interaction between health equity and dissemination & implementation science; the case of University of Colorado and University of Rochester

**DOI:** 10.1017/cts.2021.593

**Published:** 2021-03-30

**Authors:** Reza Yousefi Nooraie, Bethany Kwan, Elissa Orlando, Nancy M. Bennett

**Affiliations:** 1 University of Rochester Medical Center; 2 University of Colorado Anschutz Medical Campus

## Abstract

ABSTRACT IMPACT: This study provides a framwork to inform and organize health equity efforts and initiatives at CTSA institutes. OBJECTIVES/GOALS: to map the activities of the Clinical and Translational Science Award (CTSA) Program hubs across the EQ-DI framework, to depict opportunities for interaction between health equity and dissemination & implementation (D&I) science. METHODS/STUDY POPULATION: The EQ-DI framework demonstrates the dynamic interaction between D&I science and health equity. Health equity could be a lens to sensitize and inform D&I planning (through goal-setting and team development), execution (through ‘adaptation’ and D&I strategies), and evaluation (through incorporating health equity in D&I outcome assessment). On the other hand, D&I models, methods, and study designs can operationalize dissemination and implementation of evidence-based interventions to improve equity. Stakeholder engagement is at the center of the framework to inform and direct the sensitization and operationalization cycles. RESULTS/ANTICIPATED RESULTS: We reviewed the activities of Colorado Clinical & Translational Sciences Institute (CCTSI) and University of Rochester Clinical and Translational Science Institute (UR CTSI) to improve health equity and mapped them across the EQ-DI framework. The sensitizing activities included health equity training, eliciting community priorities, and inclusion of health equity as a critical axis in funding mechanisms. The operationalizing activities included D&I methodological training and consultation, collaborative team science, and funding mechanisms to support implementation of health equity EBIs. Community engagement through studios, community liaisons, and consults was a core priority guiding sensitizing and operationalizing activities. DISCUSSION/SIGNIFICANCE OF FINDINGS: The CTSA Program has been a champion for community engagement and translational collaboration to improve individual and population health. CTSA hubs provide infrastructure and resources to facilitate equity-focused D&I.

